# Hydrogel Systems in Plant Germplasm Cryopreservation: A Comprehensive Review

**DOI:** 10.3390/gels12020106

**Published:** 2026-01-27

**Authors:** Olena Bobrova, Viktor Husak, Alois Bilavcik, Milos Faltus

**Affiliations:** 1 Plant Physiology and Cryobiology Team, Czech Agrifood Research Center, Drnovska 507/73, 16100 Prague, Czech Republic; alois.bilavcik@carc.cz (A.B.); milos.faltus@carc.cz (M.F.); 2 Department of Cryobiophysics, Institute for Problems of Cryobiology and Cryomedicine NAS of Ukraine, Pereyaslavska 23, 61015 Kharkiv, Ukraine; 3 Department of Biochemistry and Biotechnology, Vasyl Stefanyk Carpathian National University, Shevchenka 57, 76018 Ivano-Frankivsk, Ukraine; viktor.husak@cnu.edu.ua

**Keywords:** hydrogels, encapsulation–dehydration, encapsulation–vitrification, germplasm conservation, alginate beads, cryoprotectants, cryobiotechnology

## Abstract

Cryopreservation is a critical strategy for the long-term conservation of plant germplasm, particularly for clonally propagated crops, endangered species, and plants producing recalcitrant seeds. Hydrogel-based encapsulation systems can improve survival during ultra-low-temperature storage by providing mechanical protection, moderating dehydration, and regulating cryoprotectant uptake. Although calcium–alginate beads remain the traditional matrix for encapsulation–dehydration and encapsulation–vitrification, recent advances in biomaterials science have enabled the development of composite polysaccharide blends, protein-based matrices, synthetic polymer networks, macroporous cryogels, and functionalized hybrid hydrogels incorporating surfactants, antioxidants, or nanomaterials. These engineered systems provide improved control over water state, pore architecture, diffusion kinetics, and thermal behavior, thereby reducing cryoinjury and enhancing post-thaw recovery across diverse plant explants. This review synthesizes current knowledge on hydrogel platforms used in plant cryopreservation, with emphasis on how physicochemical properties influence dehydration dynamics, cryoprotectant transport, vitrification stability, and rewarming responses. Performance across major explant types is assessed, key limitations in existing materials and protocols are identified, and design principles for next-generation hydrogel systems are outlined. Future progress will depend on material standardization, integration with automated cryopreservation workflows, and the development of responsive hydrogel matrices capable of mitigating cryogenic stresses.

## 1. Introduction

Cryopreservation has become an essential strategy for the long-term conservation of plant genetic resources, supporting global biodiversity, food security, and crop improvement efforts [1,2]. By storing biological materials at −196 °C in liquid nitrogen, cryopreservation nearly arrests metabolic activity, thereby preventing genetic instability, somaclonal variation, and the continual maintenance required for field or in vitro collections [3,4,5]. This capability is indispensable for vegetatively propagated crops, endangered species, and plants producing recalcitrant or intermediate seeds that cannot be preserved using conventional seed banking approaches. Despite broad adoption and decades of refinement, plant cryopreservation remains challenging because plant tissues, unlike isolated animal cells, contain large vacuoles, rigid cell walls, heterogeneous cell types, and high water content, all of which make them especially vulnerable to ice formation, osmotic shock, cryoprotectant toxicity, and mechanical damage during cooling and rewarming [3,5,6].

**Figure 1:**
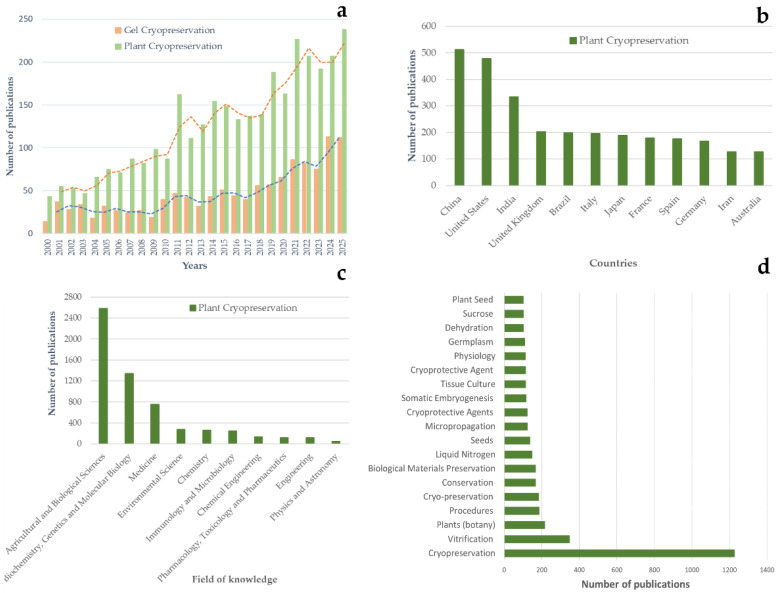
Bibliometric overview of research trends in plant cryopreservation and hydrogel-assisted cryopreservation. (**a**) Temporal distribution of scientific publications related to general plant cryopreservation and gel/hydrogel-based cryopreservation between 2000 and 2025. Bars indicate annual publication counts, while dashed lines represent trend lines illustrating overall publication growth over time. (**b**) Geographic distribution of plant cryopreservation publications by contributing countries. (**c**) Distribution of publications across major scientific subject areas. (**d**) Keyword frequency analysis based on plant cryopreservation literature, showing the most frequently used terms related to cryopreservation methods, materials, and explant types. Bar length reflects the number of publications in which each keyword appears, enabling improved readability and quantitative comparison of dominant research themes.

The rapid expansion of research in this field reflects both its importance and its unresolved challenges. A bibliometric analysis of the literature demonstrates a steady increase in plant cryopreservation publications over the past two decades (Figure 1a), with contributions spanning a wide range of countries (Figure 1b) and scientific disciplines (Figure 1c). The prominence and relative frequency of keywords such as cryopreservation, vitrification, plants, procedures, and cryoprotective agents (Figure 1d) highlight the central role of vitrification-based strategies and material-assisted approaches in plant cryopreservation research, while emphasizing sustained interest in stress control and post-thaw recovery. However, while plant cryopreservation research has expanded quantitatively, methodological success remains highly species- and genotype-dependent, indicating persistent limitations in controlling the physicochemical microenvironment during cryogenic processing.

A central goal of cryopreservation is to achieve a vitrified state in which intracellular and extracellular water solidify into an amorphous glass rather than crystalline ice [7,8]. However, achieving uniform vitrification in complex plant explants is difficult: water removal must be precisely controlled, cryoprotectants must penetrate without causing toxicity, and heat transfer must be rapid and homogeneous to avoid devitrification. Traditional liquid-based vitrification and dehydration methods often expose explants to steep osmotic gradients, uneven cryoprotectant diffusion, and uncontrolled mechanical stresses, producing highly species- and genotype-dependent outcomes. Consequently, many plant materials remain recalcitrant to existing cryotechnologies, and survival rates frequently vary unpredictably across laboratories [3,5,9].

In this context, hydrogel-based encapsulation systems have emerged as versatile platforms capable of addressing multiple cryobiological challenges simultaneously [10,11,12]. Hydrogels, three-dimensional polymeric networks with high water-binding capacity, combine liquid-like diffusion behavior with solid-like mechanical stability. As encapsulation matrices, they impose controlled dehydration kinetics, modulate cryoprotectant penetration, buffer mechanical stress, and promote more uniform heat transfer. Rather than acting as passive carriers, hydrogels actively regulate the physicochemical microenvironment experienced by plant tissues during dehydration, cooling, vitrification, and rewarming, thereby improving reproducibility and reducing cryoinjury in otherwise recalcitrant systems.

Alginate hydrogels have long dominated encapsulation–dehydration and encapsulation–vitrification protocols because of their biocompatibility, mild gelation conditions, and extensive validation in plant tissue culture [3,12,13]. Nevertheless, their limited tunability, susceptibility to dehydration-induced weakening, and batch-to-batch variability have increasingly highlighted the need for more advanced materials. Recent innovations in biomaterials science now provide expanded control over pore architecture, mechanical properties, water state, solute diffusion, and thermal behavior. These developments signal a shift from empirically optimized encapsulation toward rational, materials-driven design of cryoprotective platforms.

Despite this progress, a comprehensive synthesis linking hydrogel physicochemical properties to cryoprotective mechanisms and plant cryobiological outcomes remains lacking. Most existing reviews focus on cryopreservation protocols or synthetic seed technologies and typically treat hydrogels as passive encapsulation matrices rather than as active functional materials whose properties influence dehydration dynamics, cryoprotectant transport, vitrification stability, and post-thaw recovery. By integrating evidence from plant cryopreservation studies with mechanistic concepts from polymer science and cryobiology, while clearly distinguishing validated plant data from insights extrapolated from non-plant systems, this review aims to address that gap. We systematically analyze hydrogel systems used in plant cryopreservation, with particular emphasis on alginate-based platforms; examine how measurable material properties influence key cryogenic processes; assess performance across explant types and species; and identify research directions required for the cautious and evidence-based development of next-generation hydrogel-assisted cryopreservation strategies for global germplasm conservation.

## 2. Methodology

This review used a structured literature search and a narrative synthesis approach. The reporting logic followed established guidance for transparent evidence selection and screening [14,15].

### 2.1. Data Sources and Search Strategy

Peer-reviewed papers published in English were retrieved from Scopus, Web of Science Core Collection, ScienceDirect, PubMed, and Google Scholar. Searches were conducted using combinations of controlled terms and free text. The main keyword blocks were:

- Plant cryopreservation terms: “plant cryopreservation”, “germplasm cryopreservation”, “cryobank”, “vitrification”, “encapsulation dehydration”, “droplet vitrification”, “cryo plate”.
- Hydrogel terms: “hydrogel”, “alginate”, “calcium alginate”, “encapsulation”, “bead”, “microencapsulation”, “cryogel”.
- Explant terms: “shoot tip”, “meristem”, “somatic embryo”, “embryogenic callus”, “pollen”, “seed”, “in vitro culture”.

Boolean operators were used to connect blocks. Truncation and phrase searches were applied where supported by the database. Reference lists of key papers and relevant reviews were also screened to identify additional studies.

### 2.2. Time Window and Eligibility Criteria

This review considers literature published between 1990 and 2025, encompassing early work on encapsulation-based plant cryopreservation as well as more recent studies addressing the roles of hydrogels in cryogenic preservation. Publications were selected based on their relevance to plant cryopreservation under liquid nitrogen and the involvement of hydrogel matrices as encapsulants, carriers, or functional interfaces during cryoprocessing.

Emphasis was placed on studies reporting post-thaw outcomes, such as survival, regrowth, recovery, or viability, and providing sufficient methodological detail to identify the hydrogel systems and processing conditions used. The review focuses primarily on plant-based cryopreservation studies, while excluding reports limited to non-plant systems or hydrogel applications unrelated to cryogenic storage. Where appropriate, insights from broader hydrogel and cryobiology literature are used to support interpretation and highlight emerging research directions.

### 2.3. Screening and Study Selection

Titles and abstracts were initially screened to exclude records clearly unrelated to plant cryopreservation or to hydrogel-based encapsulation systems. The full texts of potentially relevant publications were then assessed for eligibility based on predefined inclusion criteria, including plant-based cryopreservation under liquid nitrogen and the use of hydrogel matrices during cryoprocessing. Duplicate records were removed using citation metadata and manual cross-checking. When multiple publications reported data from the same experimental series, the most comprehensive dataset was retained, while related companion papers were consulted to clarify protocols, materials, and methodological details.

### 2.4. Data Extraction and Coding

Key information was compiled from the selected studies to enable comparison across plant systems and cryopreservation approaches. Extracted details included plant material, cryopreservation procedures, and hydrogel characteristics. Information on cryoprotectant formulations, exposure conditions, and post-thaw outcomes was also considered.

To support synthesis rather than quantitative comparison, hydrogel systems were broadly grouped by polymer class and network characteristics, while cryopreservation workflows were categorized into encapsulation–dehydration, encapsulation–vitrification, or hybrid approaches. This qualitative coding was used to identify recurring patterns, functional relationships, and knowledge gaps across the literature.

### 2.5. Evidence Synthesis

A narrative synthesis was used due to heterogeneity in plant taxa, explant traits, and viability metrics. Results were compared within protocol classes and within hydrogel classes. Emphasis was placed on recurring patterns that linked hydrogel properties to cryobiological outcomes. Reported limitations and sources of variability were also summarized. This approach supported cross-study interpretation without imposing a single pooled effect estimate.

## 3. Hydrogel Systems and Their Functional Mechanisms

**Figure 2:**
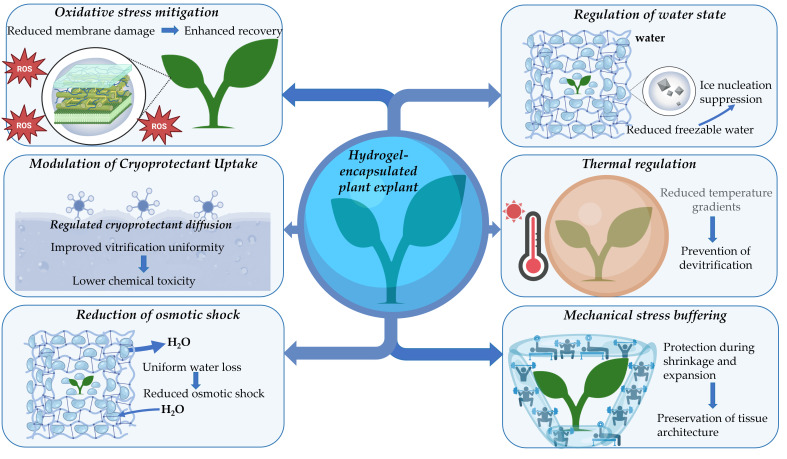
Multifunctional cryoprotective roles of hydrogel encapsulation in plant cryopreservation. Schematic representation of the key physicochemical and biological mechanisms by which hydrogel encapsulation enhances plant explant survival during cryopreservation. Hydrogel matrices surrounding the explant regulate the water state by increasing the fraction of bound water and suppressing ice nucleation, thereby reducing freezable water content. Controlled dehydration within the hydrogel network ensures uniform water loss and minimizes osmotic shock. The hydrogel structure moderates cryoprotectant diffusion, enabling more homogeneous vitrification while reducing chemical toxicity. Mechanical stress buffering protects tissue architecture during volumetric shrinkage and expansion associated with dehydration, cooling, and rewarming. Improved thermal regulation promotes thermal homogeneity and reduces temperature gradients, limiting the risk of devitrification. In parallel, hydrogel-mediated mitigation of oxidative stress reduces membrane damage and supports enhanced post-thaw recovery and regrowth.

Hydrogels used in cryopreservation belong to several material classes. These classes differ in polymer chemistry and in the routes of network formation. These differences control water retention, pore architecture, stiffness, and solute diffusion, which in turn shape dehydration kinetics and cryoprotectant transport during encapsulation-based protocols [16]. In plant cryopreservation, these structure–function relationships have been most extensively characterized for alginate-based matrices. The multifunctional roles of hydrogel encapsulation that motivate these design targets are summarized in Figure 2.

Natural polysaccharide hydrogels are the most widely used matrices in plant cryopreservation, with alginate representing the dominant and best-validated example [11,12,13,16]. Alginate gels form via ionic crosslinking with divalent cations, most often Ca^2+^. Junction zones are created by cooperative binding to guluronic acid-rich segments. This concept is usually described by the egg-box model and its later refinements based on diffraction data [17]. In plant systems, alginate beads enable gentle immobilization of meristems and embryos, and support controlled dehydration in encapsulation–dehydration methods as well as in vitrification-oriented workflows [16,18]. Alginate performance varies with polymer composition and ion exchange conditions, which alter gel stiffness and permeability. These variations influence cryoprotectant equilibration and water loss behavior, consistent with the transport and water state functions illustrated in Figure 2.

Other polysaccharide hydrogels have been explored to modify gelation behavior and water-binding characteristics, although their use in plant cryopreservation remains limited and largely exploratory. Agarose and agar gels are thermoreversible networks formed by physical association upon cooling with mechanical properties that depend on concentration and molecular features [19]. Pectin hydrogels provide additional design options. Low-methoxyl pectins can form Ca^2^-mediated junctions, while other pectin networks rely on hydrogen bonding and hydrophobic association [20]. These mechanisms change swelling and water state distribution, which is directly relevant to bound water enrichment and freezable water suppression as depicted in Figure 2. Cellulose-derived hydrogels include carboxymethyl cellulose and related derivatives. They can be crosslinked chemically or physically. They also show strong hydration and tunable porosity when solvent systems and crosslink density are controlled [21]. However, systematic plant cryopreservation studies using these matrices remain scarce, and most evidence for their behavior derives from broader hydrogel research.

Composite polysaccharide systems expand network architectures beyond single-component gels. Alginate chitosan combinations are among the most frequently reported examples. They form polyelectrolyte complexes at the interface or within blended matrices. These electrostatic interactions can increase mechanical stability and alter pore connectivity [22]. Such composite designs are relevant when beads must resist deformation during dehydration and handling while still permitting diffusion of cryoprotectants. In plant cryopreservation, however, quantitative characterization of these effects and direct performance comparisons with standard alginate systems remain limited.

Protein-based hydrogels are less common in plant cryopreservation, but they are attractive as mechanically compliant networks. Gelatin can form physically crosslinked gels and can also be chemically crosslinked to increase stability. Crosslinking level strongly affects stiffness and transport [23]. Collagen networks provide elastic fibrillar architectures. Their macroscopic mechanics depend on crosslink density and network connectivity [24]. Silk fibroin hydrogels offer robust physical crosslinking and a wide range of viscoelastic behavior. They also provide distinctive water-binding profiles and hydrogel structural stability [25]. These protein systems are relevant to future plant studies aimed at improving mechanical buffering during cooling and rewarming.

Synthetic polymer hydrogels offer high reproducibility and precise control of mesh size. Poly(ethylene glycol) networks are a reference system for relating swelling to network structure. Mesh size can be tuned by crosslink density and chain length [26]. Poly(vinyl alcohol) hydrogels are often formed by physical crosslinking of crystallites during freeze–thaw cycles. This route produces tough networks without added crosslinkers [27]. These synthetic platforms are widely used in biomedical hydrogel design, and they can support the translation of predictive structure–property concepts into plant cryopreservation. Their controlled transport properties can support reduced cryoprotectant toxicity and more homogeneous cryoprotective agent exposure, consistent with the diffusion control role in Figure 2.

Cryogels are a distinct architectural class relevant to rapid mass transfer. Cryogels form when gelation proceeds in partially frozen media. Ice crystals act as porogens, creating interconnected macropores after thawing. These pores increase permeability and accelerate solute transport [28,29]. Ice-templated chitosan-based cryogels are a representative example of how freezing can be used to tailor macroporosity and transport pathways [30]. Such architectures may support faster cryoprotectant equilibration and improved rewarming performance, but they require careful matching to dehydration needs and explant size.

Overall, hydrogel classes and network architectures define a broad design space for plant cryopreservation matrices. In practice, however, validated plant applications remain concentrated on alginate-based systems. Selection of alternative materials should be guided by the needed balance between water binding, diffusion, and mechanical support at each processing stage [16,26].

Regulating water behavior is essential for successful plant cryopreservation. Hydrogel matrices shape both the rate of water removal and the physical state of water during cooling and rewarming [11,31]. These multifunctional roles are summarized in Figure 2. Plant cells are sensitive to rapid dehydration. Steep osmotic gradients can induce plasmolysis and loss of membrane wall contacts. They can also disturb cytoskeletal organization and vacuolar structure. These effects can reduce viability after subsequent cryogenic steps [32,33,34]. Encapsulation-based methods exploit the diffusion-limited water transport imposed by hydrogels, resulting in slower and more controlled dehydration than direct air-drying or immersion in concentrated osmotic media [16,35]. This moderation is particularly important for multicellular explants with heterogeneous permeability and for shoot tips containing cells at different developmental states.

Hydrogels also shift the distribution of water states within the encapsulated system. Water in hydrogel systems can be described as free water, loosely bound water, and tightly bound water. Bound fractions show reduced molecular mobility and reduced freezing propensity. Alginate bead studies using thermal analysis quantify unfrozen water fractions and relate them to successful vitrification windows [31]. Polymer network structure influences water binding. Higher crosslink density can increase the fraction of water that is constrained. It can also slow dehydration and reduce effective water loss. Excessive constraint can therefore prevent attainment of vitrifiable water contents in the explant. This trade-off is central to encapsulation dehydration workflows and vitrification-oriented treatments [31,36].

Cryoprotectants are required to stabilize membranes and proteins and to promote glass formation. Their penetrating components can cause chemical stress and osmotic stress. Plant vitrification solution 2 provides a clear example of plant vitrification because it dehydrates and permeates tissues. It also alters freezing behavior during cryoprotection. Component-level toxicity has been quantified in mint shoot tips. These results support the need for controlled exposure kinetics [11,36,37]. Hydrogels modulate cryoprotectant transport by diffusion buffering. Mesh size and polymer chemistry set the effective diffusion path length. This can reduce acute toxicity from rapid solute influx. It can also reduce steep spatial gradients across the explant surface. This principle improves predictability in encapsulation vitrification and encapsulation dehydration protocols [16,31]. Hydrogels can also retain non-penetrating solutes such as sucrose at high local activity. This supports osmotic dehydration without forcing intracellular accumulation. Such approaches are aligned with classic encapsulation dehydration designs in which sucrose preculture and bead-mediated dehydration act together [16,35].

Mechanical stresses occur during dehydration, cooling, and rewarming. They arise from volume shrinkage, thermal contraction, and handling. Hydrogels provide viscoelastic buffering and geometric stabilization. Cooling and rewarming rates strongly influence the success of vitrification-based methods. Uneven or slow warming can permit devitrification and recrystallization. Plant-focused measurements show that faster warming and faster cooling correlate with higher survival. This principle is also emphasized in modern studies on optimizing droplet vitrification [38,39]. Hydrogels influence thermal behavior through their water content and internal structure. Uniform hydrated matrices can reduce temperature gradients between the bead and the explant. This effect supports more homogeneous thermal trajectories during both cooling and rewarming. In plant systems, thermal analysis has been used to connect dehydration status and phase transitions to survival outcomes [11,31,40].

Post-thaw recovery depends on membrane resealing and metabolic restart. Oxidative stress increases during key steps, particularly during dehydration and rapid warming. Arabidopsis seedling studies link reactive oxygen species (ROS) accumulation and antioxidant capacity to differences in survival across the protocol. Reviews synthesize ROS sources and mitigation strategies across many plant species and explant types [41,42]. Hydrogels can support post-thaw physiology by shaping rehydration kinetics and solute release. Gradual rehydration can reduce osmotic shock and secondary membrane rupture. It can also reduce ROS bursts during early regrowth. Abiotic stress preconditioning studies further support the role of antioxidant capacity in improved recovery [41,43].

The mechanisms in Figure 2 act together. Water state regulation, controlled dehydration, moderated solute diffusion, mechanical buffering, thermal mediation, and oxidative stress mitigation are interdependent. A change in crosslink density can alter both water binding and diffusion. A change in porosity can improve heat transfer but weaken dehydration control. These tradeoffs motivate mechanistic materials selection and standardized characterization in plant cryopreservation research [11,31,44].

## 4. Hydrogel Platforms for Plant Cryopreservation: Alginate and Beyond

**Figure 3:**
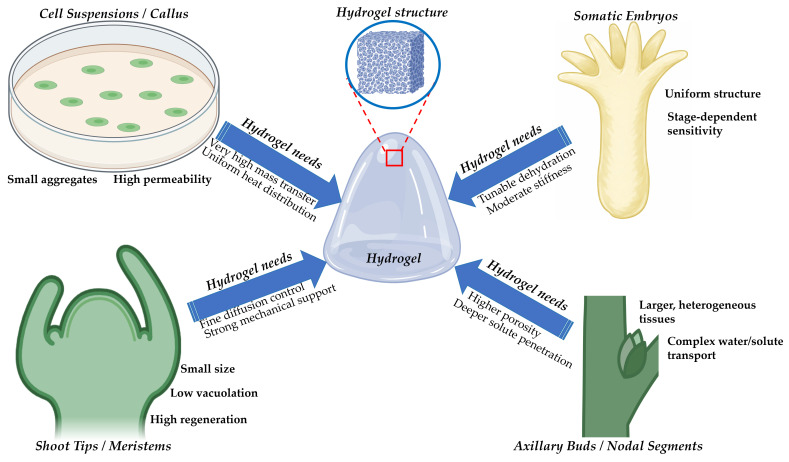
Matching hydrogel properties to plant explant types in cryopreservation. Conceptual schematic illustrating how hydrogel physicochemical properties can be tailored to the structural and physiological characteristics of different plant explant types used in cryopreservation. Cell suspensions and callus cultures require hydrogels with very high permeability, efficient mass and heat transfer, and uniform thermal distribution to support small cell aggregates. Somatic embryos, characterized by uniform morphology but strong stage-dependent sensitivity, benefit from hydrogels with tunable dehydration kinetics and moderate mechanical stiffness. Shoot tips and meristematic tissues, which are small, weakly vacuolated, and highly regenerative, require fine control of cryoprotectant diffusion combined with strong mechanical support to preserve tissue architecture. Axillary buds and nodal segments, representing larger and more heterogeneous tissues with complex water and solute transport pathways, demand hydrogels with higher porosity to enable deeper cryoprotectant penetration and more homogeneous vitrification. The central hydrogel structure highlights the tunable network architecture that enables explant-specific optimization of cryoprotective performance.

Hydrogel-assisted cryopreservation in plants has relied mainly on calcium alginate. Alginate became the standard matrix for encapsulation, dehydration, and several encapsulation-linked vitrification workflows. The material is inexpensive and easy to handle. Gelation proceeds in water and at mild temperatures. These features protect sensitive meristematic tissues during bead formation [16]. The logic for matching hydrogel properties to explant categories is shown in Figure 3. The major hydrogel material classes and representative studies are summarized in Table 1. The relationships between key hydrogel physicochemical properties and cryoprotective mechanisms across the cryogenic workflow are synthesized in Table 2. While alginate-based systems are supported by extensive plant-specific validation, most alternative hydrogel platforms discussed below remain at an exploratory or conceptual stage in plant cryopreservation, with supporting evidence often drawn from limited plant studies or from broader cryobiology and biomaterials research.

Alginate is a linear polysaccharide built from β-D-mannuronic acid and α-L-guluronic acid residues. It forms gels by ionic crosslinking with Ca^2+^. Ca^2+^ binds preferentially to guluronate-rich sequences and creates junction zones. This “egg-box” arrangement is supported by structural studies on alginate networks [17]. The gelation process is fast and gentle. It is therefore suitable for shoot tips, apical meristems, and somatic embryos.

**Table 1:** Summary of hydrogel materials used in cryopreservation, their key properties, cryoprotective functions, and representative references.

Hydrogel Material Class	Typical Polymers/Examples	Crosslinking Mechanism	Key Physicochemical Properties	Main Cryoprotective Functions	Advantages in Plant Cryopreservation	Limitations/Challenges	Key References
Alginate hydrogels	Sodium alginate (Ca^2+^-alginate beads)	Ionic crosslinking (“egg-box” model)	High water content; moderate mechanical strength; limited pore tunability	Controlled dehydration; diffusion buffering; mechanical protection	Biocompatible; mild gelation; easy handling; widely validated	Limited control of pore size; batch variability; mechanical weakening under extreme dehydration	Engelmann [45]; Sakai et al. [46]; Benelli et al. [47]; Gantait et al. [48]
Composite polysaccharide hydrogels	Alginate–chitosan; alginate–agar; alginate–pectin; cellulose blends	Ionic + hydrogen bonding/electrostatic interactions	Enhanced stiffness; adjustable porosity; improved structural stability	Improved diffusion control; enhanced mechanical buffering	Greater tunability than alginate alone; improved robustness	Increased formulation complexity; reproducibility issues	Zhang et al. [22]; Teixeira et al. [49]
Protein-based hydrogels	Gelatin; silk fibroin; collagen	Physical (thermal) or chemical crosslinking	Elastic networks; thermoresponsive behavior; moderate water binding capacity	Mechanical stress buffering; potential temperature-responsive diffusion	High elasticity; good energy dissipation	Batch variability; limited validation in plant systems	Li et al. [23]; Lin et al. [24]; Onder et al. [25]; Wang et al. [50]; Liu et al. [51]
Synthetic polymer hydrogels and cryogels	Poly(vinyl alcohol) (PVA); polyethylene glycol (PEG); polyacrylamide; composite cryogels	Chemical crosslinking or freeze–thaw	Interconnected macropores; highly tunable mesh size; high permeability; rapid swelling	Rapid solute diffusion; improved heat transfer during rewarming	High design flexibility; material reproducibility	Reduced control of water binding capacity; risk of under-dehydration	Sharma et al. [52]; Lozinsky et al. [53]; Plieva et al. [54]
Hybrid/functionalized hydrogels	Hydrogels with antifreeze proteins, antioxidants, nanoparticles, surfactants	Variable (physical/chemical)	Multifunctional networks; altered thermal and biochemical behavior	Ice inhibition; oxidative stress mitigation; enhanced stability	Active cryoprotection; multifunctionality	Increased complexity; regulatory and standardization challenges	Kulus et al. [55,56]; Yang et al. [57]; Harding et al. [58]

Note: Except for calcium–alginate matrices, most hydrogel systems listed have limited direct validation in plant cryopreservation and are included to highlight conceptual design space and future research directions rather than established cryobank practice.

Encapsulation–dehydration was introduced as a plant cryopreservation method with calcium alginate beads as the protective matrix. The approach enabled controlled water removal before direct immersion in liquid nitrogen [16,59]. Many later protocols retained alginate because the bead provides a stable geometry and improves handling. Apple shoot tips were cryopreserved using both encapsulation–dehydration and encapsulation–vitrification. The study also showed cultivar-dependent recovery [60]. Encapsulation–vitrification has also been validated for mint meristems with high shoot formation after rewarming [61]. These outcomes align with the diffusion buffering and mechanical stabilization mechanisms shown in Figure 2.

Alginate beads also provide a sound model system for studying water behavior. Differential scanning calorimetry has been used to quantify unfrozen water fractions in beads during key pretreatments. The results link bead water content to vitrification stability. The work also highlighted a need for bead standardization to reduce variability [11,31]. This mechanistic evidence supports the view that measurable physical targets should define hydrogel properties. Alginate performance is largely governed by water-binding capacity, effective mesh structure, and solute diffusion behavior. These property–mechanism links are outlined in Table 2.

**Table 2:** Relationships between hydrogel physicochemical properties and cryoprotective mechanisms in plant germplasm cryopreservation.

Hydrogel Property	Material Parameters Influencing the Property	Cryoprotective Mechanism	Cryobiological Effect on Plant Tissues	Relevant Cryopreservation Stage
Water-binding capacity	Polymer hydrophilicity; functional groups; crosslink density	Reduction of freezable water; ice nucleation suppression	Decreased intracellular and extracellular ice formation	Dehydration; cooling
Pore size/mesh structure	Polymer molecular weight; crosslinking type and density; composite formulation	Regulation of water and solute diffusion	Controlled dehydration; uniform cryoprotectant distribution	Cryoprotectant loading; dehydration
Diffusion coefficient for solutes	Network density; porosity; polymer–solute interactions	Modulation of cryoprotectant uptake kinetics	Reduced osmotic shock and chemical toxicity	Cryoprotectant exposure
Mechanical stiffness and elasticity	Polymer concentration; crosslinking strength; composite reinforcement	Mechanical stress buffering	Preservation of tissue integrity; reduced cracking and deformation	Dehydration; cooling; rewarming
Viscoelastic behavior	Polymer chain mobility; physical vs. chemical crosslinking	Energy dissipation during volume changes	Reduced mechanical damage at cellular and tissue levels	Cooling; rewarming
Thermal conductivity	Water content; density; internal architecture	Enhancement of thermal uniformity	Reduced thermal gradients; minimized devitrification risk	Cooling; rewarming
Macroporosity (cryogels)	Freezing conditions during gel formation; porogen size	Rapid mass and heat transfer	Improved cryoprotectant equilibration; faster rewarming	Cryoprotectant loading; rewarming
Swelling/deswelling behavior	Polymer chemistry; ionic strength; temperature sensitivity	Regulation of hydration dynamics	Controlled rehydration; reduced membrane stress	Rewarming; recovery
Chemical functionality	Presence of charged or reactive groups; functional additives	Interaction with water, solutes, or ice	Enhanced vitrification support; ice inhibition	Dehydration; cooling
Biochemical functionalization	Antioxidants; antifreeze proteins; osmoprotectants	Mitigation of secondary stress responses	Reduced oxidative damage; improved post-thaw regrowth	Rewarming; post-thaw recovery

Alginate has intrinsic limits that become visible in recalcitrant species and in high-throughput workflows. Natural alginate varies in its mannuronic acid-to-guluronic acid ratio, molecular mass distribution, and impurity content. These factors change stiffness, swelling, and effective diffusivity. These changes affect dehydration and cryoprotectant equilibration. They can reduce inter-laboratory reproducibility [31]. Alginate networks can also weaken during severe dehydration. Bead integrity can decrease during prolonged osmotic exposure or air desiccation.

Diffusion limits can be a second constraint. Dense beads can slow the penetration of cryoprotectants into large explants. This can create internal regions with incomplete vitrification. This issue is relevant for axillary buds and nodal segments. It is also appropriate for multicellular explants with heterogeneous permeability. These limitations explain why new platforms are being explored. They also explain why thin-layer formats and microstructured matrices are gaining attention. This trend is consistent with Figure 3. Composite polysaccharide hydrogels aim to retain alginate biocompatibility while improving robustness and transport control [55,56,57]. Composite designs often combine ionic crosslinking with additional hydrogen bonding or electrostatic interactions. Plant cryopreservation studies rarely report complete physical characterization of such composites. This gap limits direct comparison across labs.

Modified bead matrices provide a plant-focused route for improving performance without changing the main polymer. Some studies change the bead formulation by adding culture medium salts or other components. This approach can change dehydration behavior and recovery. A plant example is the evaluation of bead-matrix composition in bleeding-heart shoot-tip cryopreservation. The study compared beads based on MS medium versus water and quantified recovery after vitrification treatments [62]. Such results support the concept that the “alginate platform” includes both the polymer and bead microenvironment.

Protein-based hydrogels include gelatin, collagen, and silk fibroin. These networks can be more elastic than many polysaccharide gels. Elasticity can help buffer stresses during dehydration, cooling, and rewarming. Gelatin gels are thermoreversible and can be chemically crosslinked to increase stability. Network structure and stiffness can be tuned by concentration and crosslinking chemistry [23,24]. Silk fibroin hydrogels can provide strong physical networks and stable porosity. They also show adjustable mechanical behavior [25].

Protein hydrogels remain uncommon in plant cryopreservation. Batch variability and biodegradation can complicate protocol control. Plant tissue culture systems also have limited historical experience with these matrices. The platform remains promising for explants that need improved mechanical buffering. It is also promising for staged transport control during cooling and rewarming.

Synthetic hydrogels can offer high reproducibility and precise control of network architecture. This property is essential for standardization in cryobanks. Poly(ethylene glycol) hydrogels provide a reference framework for relating swelling and mesh size to crosslink density [26]. Poly(vinyl alcohol) hydrogels can be formed by freeze–thaw cycling. This process creates physical crystallite crosslinks and yields robust networks without added chemical crosslinkers [27]. Such matrices may resist collapse during dehydration better than some natural gels, although this advantage has not yet been systematically demonstrated in plant cryopreservation. Biocompatibility must be confirmed for each explant type. Cryoprotectant interactions must also be evaluated. The main advantage is the ability to engineer diffusion and stiffness targets that match specific tissues.

Cryogels form when crosslinking proceeds in partially frozen systems. Ice crystals act as porogens, creating interconnected macropores after thawing. These pores can increase permeability and support rapid solute exchange. They can also support efficient heat exchange. These features are valuable when protocols require fast equilibration with vitrification solutions or very rapid warming, but plant cryopreservation still lacks broad validation of cryogel matrices. Rapid warming is critical for avoiding devitrification in vitrified systems. Macroporous cryogels are therefore conceptually aligned with the thermal mediation role in Figure 2 [28]. Chitosan-based cryogels illustrate how ice templating creates large, connected pores and high permeability [30].

Plant cryopreservation still lacks broad validation of cryogel matrices. Large pores can reduce control over dehydration kinetics. They can also reduce the fraction of bound water. These trade-offs can increase the risk of intracellular ice formation if dehydration is insufficient. Cryogels may therefore require careful integration with loading solutions and dehydration steps. New hydrogel platforms increasingly incorporate functional additives targeting specific injuries. Additives include surfactants, antioxidants, antifreeze proteins, nanoparticles, and osmoprotectants. These strategies shift hydrogels from passive carriers to active cryoprotective systems. The macroporosity and thermal conductivity advantages of cryogels correspond to the rapid mass-transfer and thermal-uniformity mechanisms summarized in Table 2.

A recent example of plant integration is the incorporation of Pluronic F-68 into an alginate-based system for peach shoot tip cryopreservation. The study combined hydrogel immobilization with a surfactant and quantified improvements in survival and regrowth after vitrification and exposure to liquid nitrogen [11]. Surfactant effects on recovery have also been studied in dormant shoot tip cryopreservation systems. A persimmon study evaluated the impact of Pluronic F-68 during the recovery and regrowth steps [63]. These plant results support the idea that additives can reduce membrane-related injury and can improve post-thaw recovery.

Functionalization also raises new constraints. Additives can change diffusion and water binding. They can also introduce regulatory and biosafety questions for germplasm exchange. Standard characterization and long-term stability testing are therefore essential.

Alginate remains the benchmark hydrogel platform for plant cryopreservation. It has strong validation in encapsulation–dehydration and in encapsulation-linked vitrification workflows [16,60]. Mechanistic work on bead water status supports the use of standardized bead targets for reliable vitrification [31]. New platforms extend the design space beyond alginate. Composite gels and modified bead matrices refine transport and stability [62]. Protein and synthetic hydrogels offer tunable mechanical and diffusion properties [23,26]. Cryogels provide high permeability and improved heat transfer but require careful control of dehydration [28]. Hybrid additive systems show clear plant-level promise and motivate mechanism-driven design [11].

## 5. Performance of Hydrogel Systems Across Plant Explants and Species

Three endpoints define the performance of hydrogel-assisted cryopreservation. These endpoints are survival after liquid nitrogen exposure, regrowth into normal plants, and maintenance of genetic and epigenetic fidelity. Survival alone is not sufficient. Many tissues remain green yet fail to reestablish organized growth. Hydrogel matrices influence each endpoint through coupled control of water state, dehydration kinetics, cryoprotectant delivery, mechanical support, and thermal trajectories (Figure 2). The strength of this approach is its tunability. The limitation is that tunability creates many combinations of materials and processes. Species and genotype traits then amplify the variability (Figure 3).

Shoot tips are the most common targets in plant cryobanks. They combine genetic stability with high regeneration potential. Their response still depends on the balance between dehydration and toxicity. Alginate is used widely because gelation is mild and fast. Encapsulation also stabilizes the meristem during cutting and handling. This stabilizing effect supports the cryoprotective mechanisms summarized in Figure 2. Cryo-plate formats can further improve reproducibility. They embed shoot tips in a thin alginate layer on an aluminum plate. This format enhances heat exchange and handling. It has been validated for strawberry shoot tips and other taxa [64,65,66].

A key cause of failure in shoot tips is cryoprotectant injury. PVS2 components can be damaging in short exposures for sensitive genotypes. This effect has been documented in mint shoot tips [37]. Damage also increases at higher temperatures during exposure. Hydrogels reduce the concentration gradients at the tissue surface, resulting in the slow influx of penetrating solutes. This moderation supports more predictable vitrification. It matches the diffusion buffering role in Figure 2.

Droplet vitrification is often applied without beads. It remains relevant to hydrogel design because the method requires rapid cooling and rapid warming. Any encapsulation layer must not block heat transfer. Studies on woody fruit species show strong genotype dependence even under robust droplet protocols. Blackberry and cherry plum shoot tips illustrate this contrast. Regrowth differed strongly between the two species under similar loading and vitrification treatments [67]. Recent work also demonstrates protocol development for crops such as hops, which expands the species base used in cryobanks [68].

Axillary buds and nodal segments contain more differentiated tissues than shoot tips. They have larger vacuoles and higher free water content. They also have deeper diffusion paths. These traits increase the risk of uneven dehydration and incomplete vitrification. Dense alginate beads can limit the penetration of vitrification solutions into internal tissues. This limitation can create protected zones near the surface and underprotected zones deeper in the explant. Macroporous architectures can reduce this problem. Thin-layer cryo-plate systems are also helpful here. They reduce the diffusion distance from solution to tissue. They also improve handling consistency at scale (Figure 3). Evidence from lateral bud cryo-plate protocols supports this direction [66].

Somatic embryos have consistent geometry and high developmental competence. They are suitable for hydrogel encapsulation because encapsulation is already used in synthetic seed systems. The bead protects the embryo structure during dehydration. It also prevents physical collapse. It can also reduce shear stress during transfers. Vitrification still requires precise water control. Over-dehydration reduces viability. Under-dehydration increases ice risk. Material parameters, therefore, need to be aligned with the embryo stage. Earlier embryogenic stages often require gentler dehydration trajectories. Later stages tolerate lower water content and more substantial osmotic loads. These trends support the use of tunable composite gels and staged dehydration protocols.

Cell suspensions and callus are heterogeneous. They contain aggregates of variable size. Their permeability varies within the same culture. Encapsulation can standardize aggregate size. It can also immobilize clusters in a protective microenvironment. This reduces shear injury during loading and unloading. It also improves uniformity of solute exposure across aggregates. The method has been used for embryogenic materials in species such as grapevine. Encapsulation dehydration supported recovery and regeneration in embryogenic suspensions. Encapsulation vitrification was also demonstrated. Both approaches show that hydrogel systems can support high-value clonal material beyond meristems [69,70].

For suspension systems, water state control inside the gel is critical. Differential scanning calorimetry studies of alginate beads show how polymerization and dehydration shift the unfrozen water fraction. These measurements connect directly to vitrification stability. They also provide practical targets for bead moisture control before cooling [31].

Species-level differences arise from membrane permeability, cell wall mechanics, tissue porosity, and endogenous solute composition. Genotype effects can be equally strong within one species. These effects change the optimal match between hydrogel properties and explant traits (Figure 3). Osmotic preconditioning often improves tolerance. It can raise the baseline dehydration tolerance before vitrification. It can also stabilize cell structure. Evidence from plant cell systems shows that osmotic stress conditioning can improve tolerance to drying and cryopreservation. This supports staged dehydration strategies in hydrogel protocols [71].

Cold acclimation and antioxidant support also affect outcomes. They are especially relevant for meristem-rich tissues. Kiwifruit shoot tip work shows improved regeneration when cold acclimation is combined with sucrose preculture and antioxidant supplementation [72]. These steps reduce oxidative and structural injury during recovery. They reinforce the post-thaw stress mitigation role in Figure 2.

Regrowth quality matters. Some recovered plants show delayed growth or abnormal morphology. Molecular fidelity is also central for germplasm conservation. Genetic stability has been assessed in *Prunus* regenerated from cryopreserved apices using molecular markers. This type of evaluation is essential when new materials are introduced [73]. A broader synthesis across species indicates that most cryopreserved plants remain true to type. Variation can occur, but it is not the dominant outcome. Risk increases when tissues experience high oxidative stress or extended in vitro phases after thawing. These findings support inclusion of molecular and physiological monitoring in cryobank workflows [74].

Cryobank deployment adds constraints. Protocols must be transferable and robust. Staff skill differences can change outcomes. Hydrogel systems can improve handling and reduce direct tissue damage. They still require standardization of bead size, polymer concentration, and crosslinking time. Practical cryobank implementation studies emphasize this need. They also highlight the importance of consistent regrowth testing and documentation [75].

The evidence supports a matching strategy rather than a universal gel. Figure 3 summarizes this concept at the explant level:

- Meristems benefit from moderate diffusion resistance and strong mechanical buffering.
- Somatic embryos benefit from collapse-resistant networks and stable dehydration behavior.
- Nodal systems benefit from architectures that improve deep solute penetration.
- Cell suspensions benefit from improved permeability and heat-transfer efficiency.

These targets align with the multifunctional roles in Figure 2. Material changes will shift several mechanisms at once. This coupling should be treated as a design constraint. It should also be treated as an opportunity for optimization across diverse plant germplasm.

## 6. Challenges, Limitations, and Standardization Issues

**Figure 4:**
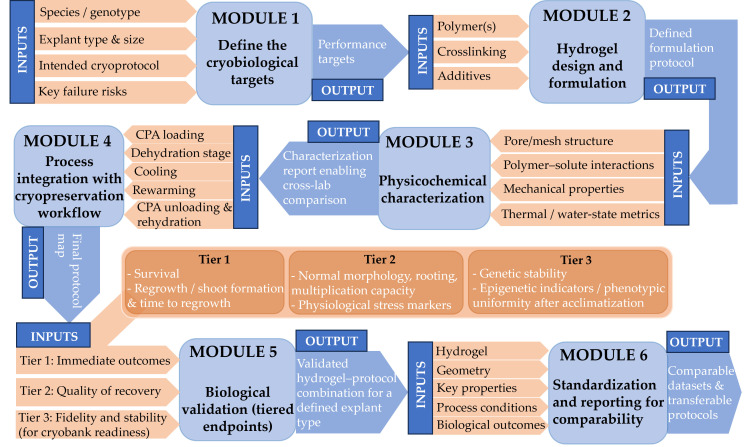
Proposed workflow for the rational development and validation of hydrogel-based cryoprotectants for plant germplasm cryopreservation. The scheme links (i) explant- and protocol-specific requirements, (ii) hydrogel formulation and geometry, (iii) minimum physicochemical characterization (pore/mesh structure, polymer–solute interactions and diffusion, elastic modulus and viscoelastic behavior, and recommended thermal and water-state metrics), (iv) integration into cryoprocessing steps, and (v) tiered biological validation, including regrowth quality and genetic stability. The final reporting checklist is intended to improve inter-study comparability and protocol transferability.

The challenges outlined above emphasize the need for greater standardization and mechanistic rigor in hydrogel-assisted plant cryopreservation. To address these issues, Figure 4 presents a structured development workflow that integrates standardized material characterization, protocol definition, and tiered biological validation, linking key physicochemical parameters to cryoprocessing steps and downstream biological outcomes.

Hydrogel-assisted cryopreservation has advanced the conservation of plant germplasm. Many constraints still limit routine use across large cryobanks. These constraints arise from hydrogel variability and from biological variability in plant tissues. They also arise from gaps in reporting and benchmarking. Addressing these issues is required for reliable technology transfer and for scalable cryobanking.

Natural hydrogels remain dominant in plant encapsulation workflows. Alginate is the primary example. Alginate properties depend on the mannuronic-to-guluronic acid ratio and on ionic crosslinking conditions. These parameters change between suppliers and between batches. They change gel stiffness, pore structure, swelling, and ion exchange behavior [76]. Such variation shifts dehydration kinetics and cryoprotectant exposure in the same nominal protocol. It reduces reproducibility across laboratories.

Other polysaccharide matrices also show strong composition dependence. Agarose mechanics depend on molecular weight and polymer structure, which can vary with processing and source material [19]. Agar gels also show composition–rheology links that complicate cross-lab equivalence when “agar” is reported without a specification [77]. Pectin gels depend on the degree of esterification and extraction chemistry, which affects charge density and network formation [78,79]. These factors support the need for tighter material specifications in cryopreservation studies.

Many plant studies still report hydrogel formulations only by polymer and crosslinker concentrations. This is not sufficient for mechanistic comparison. Diffusion in hydrogels depends on mesh size and polymer–solute interactions. It also depends on heterogeneity within the network [26]. Calcium alginate bead structure and diffusion behavior vary with bead diameter, alginate concentration, and gelation conditions [80]. Calcium introduction mode also changes internal structure and diffusion pathways, even at identical bulk composition [81]. These effects can alter cryoprotectant penetration and removal.

A practical limitation is the lack of routine reporting of elastic modulus, viscoelastic behavior, swelling ratio, and effective diffusion coefficients. Another limitation is the limited reporting of the water state. Thermal analysis can detect freezable water and glass transitions in alginate beads that are used in plant protocols [31,82]. These measurements are rarely adopted as standard controls. This gap slows protocol transfer and prevents meaningful meta-analysis.

Encapsulation–dehydration and encapsulation–vitrification are widely used. They are often optimized empirically for a single genotype and a single explant type. Standard descriptions exist, but outcomes still vary with local conditions [83]. Slight differences in bead size, crosslinking time, sucrose loading schedules, and air-flow dehydration can change final water content. They also change chemical stress during cryoprotectant exposure. Many protocols do not specify humidity, airflow velocity, or bead production method. This creates operator dependence.

Even when nominal parameters match, outcomes can diverge after transfer to a new laboratory. Species-specific responses also dominate. Encapsulation–dehydration studies in horticultural and woody taxa illustrate the need for repeated optimization when tissue size, cuticle barriers, or regeneration routes differ [84]. This variability is a significant barrier to harmonized cryobanking.

Hydrogels regulate water and solute transport. This is beneficial, but it introduces new failure modes. Dense or highly crosslinked gels can slow the diffusion of cryoprotectants and create gradients. Gradients increase local under-protection and local toxicity. Diffusion limits are well established in hydrogel transport theory [26]. They are also documented for calcium alginate systems where structure controls diffusion rate [80].

High-porosity materials can have the opposite problem. They can fail to restrict free water sufficiently. This increases the risk of intracellular ice. There is also a trade-off between permeability and dehydration control. Plant protocols rarely quantify these trade-offs. This limits rational scaling to larger explants such as nodal segments or complex meristems.

Rewarming rate is a significant determinant of vitrification success. Slow warming promotes devitrification and recrystallization. This principle is well supported in cryobiology theory and experiments [85,86]. It is also supported by data showing strong effects on warming rates in vitrified systems [87]. Hydrogels can influence heat transfer by adding thermal mass and sustaining gradients. Differential scanning calorimetry can resolve thermal events in alginate beads during plant protocols, but these measurements are not yet routine controls [31,82].

This issue becomes more critical for advanced methods. Droplet vitrification and cryo-plate approaches rely on rapid cooling and rapid warming. They reduce thermal resistance by using small volumes and conductive supports. They still require correct matching of hydrogel geometry to the warming workflow [39,88]. A mismatch can negate the benefit of ultra-rapid warming.

Many published workflows rely on manual bead extrusion and manual handling. This approach is not compatible with cryobanks that process thousands of accessions. Scaling introduces new variance sources. Bead size distribution widens. Crosslinking uniformity decreases. Batch processing increases time in intermediate states. These factors alter water content and solute exposure.

Cryo-plate methods show a route toward standardization and handling efficiency. They embed shoot tips in small volumes of alginate within standardized supports. They also simplify transfers between solutions [89,90]. Even so, plate fabrication, alginate loading, and dehydration timing require strict control. Similar needs apply to droplet vitrification workflows that depend on droplet volume and foil handling [39,88]. Large-scale deployment, therefore, needs validated fabrication protocols and quality-control checkpoints.

Hydrogel matrices can change local cryoprotectant composition. They can absorb solutes and alter activity. They can also delay equilibration during loading and unloading. This is critical for vitrification solutions with narrow safety margins. It is also critical for rapid loading schedules in droplet vitrification and cryo-plate methods [39,89]. Without diffusion measurements and standardized exposure schemes, results remain difficult to generalize.

Hydrogels can undergo ion exchange and network relaxation. These effects can occur even at low temperatures. They can change bead integrity during storage and handling. Functional additives raise additional issues. Nanoparticles, surfactants, and bioactive ice inhibitors require clear safety and traceability. This is important for international germplasm exchange. It is also essential for long-term genetic resource stewardship. Quality systems in cryobanking, therefore, need defined acceptance criteria for new hydrogel components.

Quantitative links between hydrogel properties and biological outcomes remain scarce in plant systems. Heat and mass transfer modeling has advanced in cryobiology for tissues and constructs, but its translation to plant hydrogel–explant systems remains limited [91]. A key gap is the lack of predictive tools that combine diffusion, dehydration, and thermal profiles with explant physiology. Without such tools, hydrogel selection remains empirical.

Progress toward global adoption requires coordinated standardization. Key needs include:

- Reference hydrogels and reporting standards for modulus, swelling ratio, bead geometry, and diffusion proxies [26,80].
- Standard thermal metrics for freezable water and vitrification behavior in encapsulation formats [31,82].
- Benchmark protocols and hardware for bead production and cryo-plate handling, with precise tolerances for timing and volumes [89,90].
- Cross-laboratory validation using shared genotypes and shared material lots to separate biological variance from materials variance [83].
- Warming-rate control guidelines aligned with devitrification risk, especially for vitrification-based workflows [85,86].

These actions will reduce hidden variability and improve transferability. They will also support automation and high-throughput cryobanking. They are necessary steps to make hydrogel-assisted plant cryopreservation a standardized conservation technology.

## 7. Future Perspectives and Research Directions

Hydrogel-assisted cryopreservation is moving from empirical practice to materials-led engineering. This shift is essential for cryobanking at scale and for reproducible outcomes across taxa [3]. Encapsulation systems based on alginate already show substantial operational value in plant cryopreservation and storage workflows [35,84]. Future progress will depend on linking hydrogel design variables to specific cryobiological constraints in each protocol class, including vitrification and encapsulation–dehydration [3,44].

A primary goal is to design hydrogels with a defined pore structure, water-binding capacity, and transport behavior. The water state in hydrogels can be divided into non-freezing, freezing-bound, and free water fractions. These fractions can be quantified with thermal analysis and linked to polymer chemistry [92,93]. This knowledge can guide the development of matrices that reduce freezable water without causing damaging dehydration. It can also guide matrices that control the rates of cryoprotectant entry and exit.

Diffusion is a central constraint in hydrogel–tissue systems. Solute transport depends on mesh size, tortuosity, and polymer–solute interactions [26,94]. Future hydrogel platforms should target the diffusion needs of explants. Shoot tips, embryogenic callus, and somatic embryos differ in size and permeability. They also differ in mechanical sensitivity. Donor plant status and tissue vigor also change stress tolerance and recovery, so hydrogel design should be tested with controlled donor conditions [39].

Smart hydrogels can change swelling, permeability, or stiffness in response to temperature and other cues. Thermoresponsive gels are well described in polymer science and drug delivery research [95,96]. These concepts can be translated to plant cryopreservation. A thermoresponsive matrix can support staged loading and unloading of cryoprotectants. It can also modulate dehydration kinetics during cooling and early rewarming.

Self-healing hydrogels can recover network integrity after damage. This property is relevant during bead handling, thermal contraction, and rapid cooling steps [97]. Mechanically adaptive hydrogels are also promising. A stiffer network during cooling can limit deformation and protect fragile meristem regions. A softer network after rewarming can support expansion and regrowth.

Future matrices will likely combine physical encapsulation with active cryoprotection. Ice inhibition strategies include antifreeze proteins, synthetic ice recrystallization inhibitors, and hydrogel-based anti-icing concepts [98,99]. Antifreeze proteins can provide substantial control over ice growth, but cryoprotection is not solely explained by inhibition of ice recrystallization in many systems. This point supports the need for multiparameter designs that include transport and toxicity control [100].

Oxidative stress is a common pathway of damage during dehydration, cooling, and rewarming in plants. Reactive oxygen species can increase during these phases, limiting regrowth. Antioxidant strategies and redox management are therefore essential targets for hydrogel delivery and local buffering [41,42]. Sustained-release antioxidant hydrogels are a practical direction because they can reduce peak exposure while maintaining protective levels during recovery.

Nanoparticle-enhanced systems are another route to multifunctionality. Nanoparticles can modify thermal conductivity and heat transfer behavior. This can support more uniform cooling and rewarming conditions in small-volume formats [101]. Any nanoparticle strategy must be screened for plant tissue compatibility and for downstream handling constraints in cryobanks.

Global cryobanking needs scalable fabrication and consistent material performance. Encapsulation–dehydration is already compatible with routine handling and batch workflows, but bead variability remains a challenge [35,84]. Microfluidic production of monodisperse alginate microgels offers a route to uniform bead size and uniform mass transfer conditions [102]. Robotic dispensing and standardized gelation chemistries can further reduce operator effects.

Ultra-rapid cooling methods are expanding in plant systems, including droplet-based approaches. These methods can benefit from thin-film or microbead hydrogels that facilitate rapid heat transfer while maintaining explant positioning and control of hydration [68]. Future materials should be designed for compatibility with automated liquid nitrogen handling and with rapid rewarming tools. They should also support imaging-based viability assessment in closed or semi-closed formats.

Mechanistic models can reduce the need for trial-and-error protocol tuning. Hydrogel–explant systems are suitable for coupled modeling of diffusion, dehydration, heat transfer, and mechanical stress. Transport limitations and cryoprotective agent toxicity can be integrated into predictive frameworks for protocol design [103,104]. Imaging tools such as cryomacroscopy can provide direct observations of vitrification, crystallization, and fracture events in cryoprotectant systems. These observations can inform model validation and material selection [105,106].

Machine learning can complement mechanistic modeling. It can map complex design spaces with many interacting variables, including polymer composition, pore size, loading times, and cooling rates. Successful demonstrations exist in cryopreservation media optimization and algorithm-driven protocol design [107,108]. Similar strategies can be adapted to plant cryopreservation datasets. The key requirement is standardized metadata for explant type, donor state, and hydrogel properties.

Sustainability and stewardship should guide the development of new hydrogel systems. Biodegradable and renewable polymers can reduce environmental impact and simplify waste management. Low-toxicity formulations can also support international exchange and regulatory alignment. Long-term stability in cryogenic storage is also critical. Materials should not undergo harmful ion exchange or structural drift during prolonged storage. These interconnected design, characterization, integration, and validation steps are summarized in the proposed workflow for rational hydrogel development and assessment shown in Figure 4.

Genetic and developmental fidelity remain core outcome metrics. Some systems show that cryopreservation can affect growth traits without disrupting chimeric structure in ornamental materials. This observation supports careful phenotypic and molecular monitoring when new materials are introduced [109]. Future hydrogel platforms should therefore be evaluated with multi-year regrowth studies and with genetic and epigenetic assays that match the crop and tissue type.

Design-driven materials and quantified performance will define the next generation of hydrogel-assisted plant cryopreservation. Hydrogels should be selected and engineered using water state control, diffusion modeling, and mechanical matching to explant needs [26,93]. Innovative and multifunctional matrices can reduce cellular stress during cooling and rewarming and may offer opportunities to improve recovery consistency [96,98]. High-throughput cryobanking will require standardized fabrication and data-rich validation across species and collections [3,102].

## 8. Conclusions

Hydrogel-assisted cryopreservation represents a versatile and increasingly important strategy for the long-term preservation of plant germplasm, particularly for vegetatively propagated species and materials that are recalcitrant to conventional seed storage. Encapsulation systems, most notably calcium–alginate matrices, can improve cryopreservation outcomes by regulating dehydration, moderating cryoprotectant transport, buffering mechanical stress, and promoting more uniform thermal behavior during cooling and rewarming. Beyond traditional alginate beads, emerging composite, protein-based, synthetic, macroporous, and functionalized hydrogels expand the design space for tailoring cryoprotective microenvironments to explant-specific physiological and structural requirements. Linking hydrogel physicochemical properties to cryobiological mechanisms supports a shift from empirically optimized protocols toward more rational, materials-driven cryopreservation strategies.

For cryobank implementation, broader adoption of hydrogel-based systems will depend on improved standardization, reproducibility, and scalability. Variability in hydrogel composition, limited physicochemical characterization, and inconsistent reporting remain key barriers to protocol transfer across laboratories. Addressing these challenges will require harmonized reporting standards, routine integration of material metrics, and systematic cross-species validation. Advances in engineered and responsive hydrogels, together with automation and high-throughput cryopreservation workflows, offer significant opportunities to enhance reliability, operational efficiency, and long-term security of global plant germplasm collections.

## Data Availability

No new data were created or analyzed in this study. Data sharing does not apply to this article.
